# How can we consider variable RBE and LET_d_ prediction during clinical practice? A pediatric case report at the Normandy Proton Therapy Centre using an independent dose engine

**DOI:** 10.1186/s13014-021-01960-w

**Published:** 2022-02-04

**Authors:** Stewart Mein, Benedikt Kopp, Anthony Vela, Pauline Dutheil, Paul Lesueur, Dinu Stefan, Jürgen Debus, Thomas Haberer, Amir Abdollahi, Andrea Mairani, Thomas Tessonnier

**Affiliations:** 1 Division of Molecular and Translational Radiation Oncology, National Center for Tumor Diseases (NCT), Heidelberg University Hospital, Heidelberg, Germany; 2 Heidelberg Institute of Radiation Oncology (HIRO), German Cancer Research Center (DKFZ), Heidelberg, Germany; 3 German Cancer Consortium (DKTK), Heidelberg, Germany; 4 Clinical Cooperation Unit Radiation Oncology, German Cancer Research Center (DKFZ), Heidelberg, Germany; 5 Present Address: Heidelberg Ion-beam Therapy Center (HIT), In Neuenheimer Feld (INF) 450, DE 69120 Heidelberg, Germany; 6 Faculty of Physics and Astronomy, Heidelberg University, Heidelberg, Germany; 7 Radiation Oncology Department, Centre François Baclesse, Caen, France; 8 Radiation Oncology Department, Centre Guillaume Le Conquérant, Le Havre, France; 9 ISTCT UMR6030-CNRS, CEA, Université de Caen-Normandie, Equipe CERVOxy, Caen, France; 10 National Centre of Oncological Hadrontherapy (CNAO), Medical Physics, Pavia, Italy

**Keywords:** Proton therapy, Dose calculation, LET, Treatment planning, GPU, Pediatrics

## Abstract

**Background:**

To develop an auxiliary GPU-accelerated proton therapy (PT) dose and LET_d_ engine for the IBA Proteus®ONE PT system. A pediatric low-grade glioma case study is reported using FRoG during clinical practice, highlighting potential treatment planning insights using variable RBE dose (D_vRBE_) and LET_d_ as indicators for clinical decision making in PT.

**Methods:**

The physics engine for FRoG has been modified for compatibility with Proteus®ONE PT centers. Subsequently, FRoG was installed and commissioned at NPTC. Dosimetric validation was performed against measurements and the clinical TPS, RayStation (RS-MC). A head patient cohort previously treated at NPTC was collected and FRoG forward calculations were compared against RS-MC for evaluation of 3D-Γ analysis and dose volume histogram (DVH) results. Currently, treatment design at NPTC is supported with fast variable RBE and LET_d_ calculation and is reported in a representative case for pediatric low-grade glioma.

**Results:**

Simple dosimetric tests against measurements of iso-energy layers and spread-out Bragg Peaks in water verified accuracy of FRoG and RS-MC. Among the patient cohort, average 3D-Γ applying 2%/2 mm, 3%/1.5 mm and 5%/1 mm were > 97%. DVH metrics for targets and OARs between FRoG and RayStation were in good agreement, with ∆D_50,CTV_ and ∆D_2,OAR_ both ⪅1%. The pediatric case report demonstrated implications of different beam arrangements on D_vRBE_ and LET_d_ distributions. From initial planning in RayStation sharing identical optimization constraints, FRoG analysis led to plan selection of the most conservative approach, i.e., minimized D_vRBE,max_ and LET_d,max_ in OARs, to avoid optical system toxicity effects (i.e., vision loss).

**Conclusion:**

An auxiliary dose calculation system was successfully integrated into the clinical workflow at a Proteus®ONE IBA facility, in excellent agreement with measurements and RS-MC. FRoG may lead to further insight on D_vRBE_ and LET_d_ implications to help clinical decision making, better understand unexpected toxicities and establish novel clinical procedures with metrics currently absent from the standard clinical TPS.

## Background

Proton therapy (PT) administers high-precision dose in solid tumors and potentially minimizes risk of adverse effects in nearby healthy tissues compared to photons [1, 2]. Each year, the number of centers equipped with proton beams for patient treatment is increasing, most of which involve sophisticated active beam scanning delivery for highly conformal distributions [3]. Aside from general knowledge of the biophysical implications of proton beams in terms of conventional endpoints, i.e., dose, linear energy transfer (LET) and tissue type, clinical protocols may be limited in scope and tools beyond what is currently capable by the standard clinical treatment planning system (TPS). That said, by no means is the current state of the clinical TPS not powerful—these systems offer sophisticated physics engines, optimization algorithms and approaches to planning robust intensity modulated proton therapy (IMPT) treatments considering various patient set-up and range uncertainties [4–6]. Nonetheless, quantitative biophysical considerations beyond the existing clinical assumption of a constant relative biological effectiveness (RBE) of 1.1, are not yet making impact on the clinical workflow—and for good reason. Relating proton-to-photon prescription doses and organ at risk (OAR) constraints, the current assumption of fixed RBE is supported by decades of clinical outcome towards conservative tumor control. Despite extensive knowledge and experimental evidence of enhanced biological effect (RBE > 1.1) increasing towards the distal-end within the Bragg peak [7–10], in-patient correlations of RBE enhancement remain unclear, indirect or partial [11]. Similarly, few works present potential evidence of increased toxicity with high LET [12].

Recent efforts to elucidate clinical implications of LET and RBE, and establish clear motivations/guidelines discuss present and future use of LET and evidence-based variable RBE models [13]. More specifically, the authors of the TG-256 report regarding RBE in PT recommend that the community maintains current clinical practice with constant RBE but for specific scenarios adapts clinical practice to account for potential impact of elevated RBE. In other words, potential changes in handling RBE must not reduce physical dose in tumor or increase physical dose in specified volumes of normal tissues. Moreover, the authors advocate large-scale assessment of treatment planning and delivery based on RBE-weighted dose (D_RBE_) and LET related to clinical outcome and toxicity. These goals are further discussed in a recent outlook of the future for PT and current needs to improve clinical practice with RBE [14].

To this end, clinical integration and validation of auxiliary systems are needed to provide independent calculations for both advanced biophysical computations and support during routine QA. Several institutions present development and validations of facility-specific dose engines, both Monte Carlo (MC) codes and analytical algorithms, many of which involve task parallelization on a graphics processing unit (GPU) for enhanced accuracy and speed with respect to conventional systems [15–17]. FRoG, for example, approaches these shortcomings of the clinical TPS by providing an open-architecture, GPU-accelerated analytical dose engine, capable of full patient calculations within minutes [18–20].

At the moment, there is no generalized or streamlined solution to make such computations available to the particle therapy clinic, e.g., dose-averaged LET (LET_d_) and variable RBE models and next-generation beam-models for novel treatments and delivery techniques [21–23]. This is a set-back particularly for smaller clinics which may lack time and resources necessary to allocate dedicated tools and research teams to work beyond clinical practice. Joining a list of 25 facilities invested in IBA Proteus®ONE solutions (Ion Beam Applications SA, Louvain-la-Neuve, Belgium), the Normandy Proton Therapy Center (NPTC, CYCLHAD) at the Centre François Baclesse (CFB) started patient treatment in Q3/2018, providing PT treatments using the RayStation® TPS (RaySearch, Stockholm, Sweden).

In this context, at NPTC, FRoG was established, commissioned, and verified against the clinical TPS, RayStation MC (RS-MC) to offer an auxiliary system for investigating advanced treatment design using biophysical metrics and LET computation. With FRoG, physicians and physicists can readily estimate “delivered biological dose” in critical clinical cases where fixed-RBE assumptions are subject to scrutiny. In the literature, aside from integration of independent dose engines for patient QA or retrospective analysis [24–27], works have yet to present how these systems can make tangible impacts in the live clinical workflow to adapt treatment planning based on secondary RBE and/or LET metrics.

Here, we present the development and validation of the FRoG dose engine at an IBA Proteus®ONE facility. A pediatric ocular nerve low-grade glioma case study is reported where FRoG was used prospectively during clinical practice, highlighting potential insights using LET_d_ and variable RBE as indicators for clinical decision making at existing and upcoming centers.

## Methods

The development, installation, commissioning and clinical application of FRoG for IBA Proteus®ONE facilities are outlined in the following sections.

### FRoG: from initial development to a physics model for Proteus®ONE

The FRoG system was initially developed for GPU-accelerated dose calculation for light and heavy ions at synchrotron-based facilities, the Heidelberg Ion-beam Therapy Center (HIT, Germany) and the Centro Nazionale di Adroterapia Oncologica (Italy) [18, 19, 28, 29]. Parallelization of the pencil beam (PB) algorithm in FRoG offers within a single dose kernel execution on the GPU physical dose, LET_d_ and D_RBE_ applying various biological models and parameters as well as robustness analysis for supporting research and clinical activity. Previous reports detail extensive validations of FRoG against gold-standard FLUKA MC simulation and/or reference dosimetric measurements for dose and LET_d_ [18, 19, 28]. Most recently, FRoG was implemented at a ProBeam® (Varian, Palo Alto, USA) facility for support as a secondary dose engine [20]. Initially, development involved python/C++ programming in FRoG to appropriately handle cyclotron-based energy selection (continuous) as opposed to synchrotron-based energy selection (discrete), and facility/vendor-specific DICOM formats, followed by beam-model development and validation procedures. Here, the FRoG partnership with NPTC extends functionality for IBA’s Proteus®ONE.

In addition to previous modifications made for the Varian ProBeam® facility, the FRoG dose engine was updated according to specific requirements of the Proteus®ONE system. For example, differences in continuous energy selection (cyclotron-based delivery) and consequent beam characteristics were considered to model treatment room and vendors specifications. More specifically, FRoG beam-model was generated for NPTC-specific parameters: energies, beam modifiers and spot size. The relatively thick range shifter (RaShi) used at NPTC (~6.5 cm Lexan, with water-equivalent thickness of ~7.4 cm) required a higher order Gaussian beam-model which was parameterized using a triple Gaussian to best describe the lateral dose evolution in water and the low-dose envelope (secondary beam profile from scattering in the nozzle and beam modifiers). Furthermore, while the FRoG dose engine was previously designed for a single virtual source axis distance (VSAD), a double virtual source (where VSAD_x_ ≠ VSAD_y_) implementation was necessary for the Proteus®ONE system. Detailed descriptions of the beam-model for physics and biophysical calculations are provided in the *Appendix*.

### Beam-model validation

For all commissioned energies, forward calculations of the 10.4 × 10.4 cm^2^ iso-energy layer (IEL) plans were performed to verify range and absolute dose beam calibration between FRoG, RS-MC and measurements (PPC05, IBA Dosimetry).

Similarly, a set of spread-out Bragg peak (SOBP) plans used during facility commissioning were optimized for 2 Gy target dose and subsequently calculated in FRoG and RS-MC for comparison with measurements. The SOBP plans ranged in field size (3 × 3 × 3 cm^3^, 6 × 6 × 6 cm^3^, 10 × 10 × 10 cm^3^) and depth (5 to 25 cm). Tests for shallow target depths (<10 cm) applied the RaShi while mid-range to deep-seated targets were without RaShi. Lastly, comparison of calculation performances in homogenous and heterogenous scenarios using an anthropomorphic head phantom (CIRS PT Dosimetry Head, Model 731-HN) was performed and outline in the *Appendix*.

### Patient verification

Following development and physical validations, 9 brain and base-of-skull patient cases were collected. These indications are representative of ~ 95% of patient treatments at the facility. Specific details regarding each patient case (e.g. prescription dose, CTV volume, etc.) are provided in Table 1. The cohort included various disease types such as glioma, meningioma, ependymoma, adenoma and neurinoma. Clinical single field optimization (SFO) patient plans optimized and calculated in RS-MC assuming fixed RBE = 1.1 were forward calculated in FRoG. To test agreement between RS-MC and FRoG, DVH analysis was performed in RayStation and subsequently 3D gamma (3D-Γ) analysis [31] was performed in Verisoft (PTW, Freiburg, Germany) using dose difference and distance-to-agreement criteria of 2%/2 mm, 3%/1.5 mm, 5%/1 mm with a 10%, 50% and 90% dose threshold (DT). DVH analysis took place for relevant structures i.e. CTV, chiasma, brainstem, and optic nerves, for standard metrics such as D_RBE_ and LET_d_ to X% of the structure’s volume (D_x_ and LET_x_) for 98%, 50%, 2% and 1% of the volume.

**Table 1:** Patient treatment case information

Patient	Prescription dose [GyRBE]	CTV volume (cm^3^)	Number of PBs	# of beams total	# of beams w/RS	RaShi mean distance (mm)
A	54.0	45.0	1275	2	2	70-80
B	52.2	215.0	7929	2	2	69-54
C	54.0	90.0	2936	2	2	52-46
D	54.0	87.0	2867	2	2	50-45
E	54.0	9.0	981	2	0	–
F	52.2	18.4	921	2	2	71-72
G	50.4	6.0	882	2	1	68
H	59.4	59.0	1858	2	2	56-51
I	59.4	37.0	1457	2	2	49-51

### Pediatric case study

Following validation and commissioning, FRoG was employed to support clinical decision-making for challenging patient treatments. A pediatric case exhibiting optic nerve low-grade glioma (Patient A in Table 1) was identified during routine treatment planning. This case presented concerns regarding preservation of the contralateral optic nerve (located in proximity of the target near the boundaries of the CTV) and vision. Five treatment options (Tx.#1-#5) varying in number of beams and selected beam angles were optimized in RayStation with a prescription dose of 54GyRBE in the target volume and fulfilling constraints on maximum D_0.03 cc_ in the left optic nerve and chiasma of 52GyRBE. All five plans met clinical standards for target coverage and preservation of the contralateral optic nerve in terms of the clinical RBE = 1.1 scheme.

In addition to the clinical protocol, supplementary evaluations were performed using FRoG as an independent dose engine for treatment selection in an effort to minimize variation in D_RBE_ between fixed and variable RBE as well as reducing high-LET components in the contralateral optic nerve. Plans were subsequently forward calculated in FRoG for LET_d_ and biologically weighted dose (D_RBE_) applying the variable RBE model described in McNamara et al. (vRBE_MCN_) with (α/β)_x_ = 2 Gy [32]. Together with the 5 original RS-MC optimized plans, FRoG LET_d_ and D_RBE_ distributions were analyzed within RayStation for subsequent selection by the clinical team of the optimal plan regarding the end-points of interest.

## Results

Following development and modification of FRoG for IBA Proteus®ONE facilities, simple tests to verify the FRoG physics engine were performed via calculation and comparison with RS-MC as the reference. The *Appendix* presents an overview of the beam-model, ray tracing and subsequent dose calculation for the 16-spot grid plan for evaluating the VSAD implementation specific to the Proteus®ONE system. Dose maps and central line profiles along the x-axis and y-axis are presented for RS-MC and FRoG for the lowest and highest commissioned energies (98 MeV and 226 MeV). Analysis was conducted to verify beam propagation (beam positioning and dose evolution) from the entrance channel (EC) to the Bragg peak (BP). Overall, mean deviations in position and FWHM between FRoG and RS-MC were sub-millimeter on the order of < 0.1 mm.

**Fig. 1:**
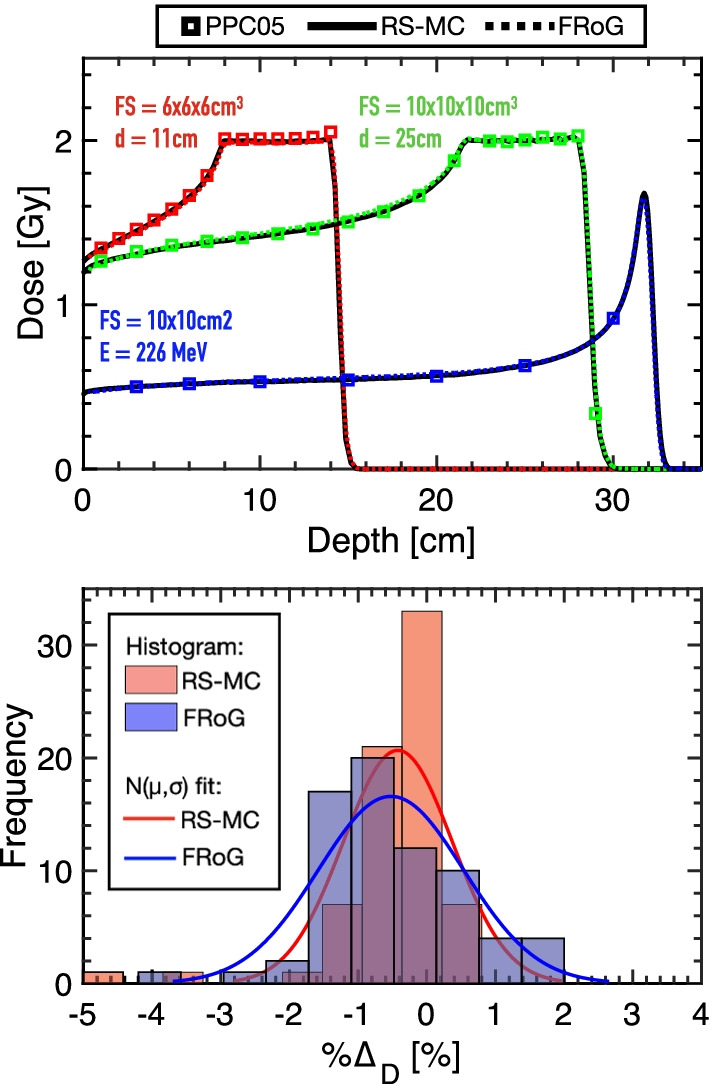
Representative data from commissioning and validation of NPTC, displaying physical dose measurements in iso-energy layers (IELs) and spread-out Bragg peaks (SOBPs) for RS-MC versus FRoG predictions (top). Percentage dose difference from measurement to FRoG (blue) and RS-MC (red) prediction presented as a histogram alongside fittings for each dataset with a normal distribution (N[µ,σ]) (bottom)

Results for representative IEL and SOBP predictions for FRoG and RS-MC against measurements are displayed in Fig. 1. IEL calibration plans were calculated in FRoG and RS-MC, yielding a mean percent difference ($\% {{\Delta}} _{D}$) of − 0.29 (± 0.39)%. Against measurements with PPC05, FRoG and RS-MC predictions were in agreement within ~ 0.6%, with $\%{{\Delta }}_{D,FRoG}$ and $\%{{\Delta }}_{D,RS-MC}$ of 0.32 (± 0.52)% and 0.13 (± 0.22)%, respectively. Differences in predicted range (∆R80) between FRoG and RS-MC was ⪅0.5 mm.

For the SOBPs optimized for 2 Gy target dose, FRoG and RS-MC predictions were in agreement with mean percent difference of 0.12(± 0.28)%. Results for absolute dose measurement with PPC05 in water against RS-MC and FRoG prediction yielded absolute mean percent differences of 0.53% and 0.96%, respectively. The histogram data provided in Fig. 1 represent percent dose difference (%∆) for SOBP plan measurement versus prediction. Fitting the data distribution with a normal function yields µ(± σ) values of −0.42(± 0.79) and −0.52(± 1.06)% for RS-MC and FRoG, respectively.

**Fig. 2:**
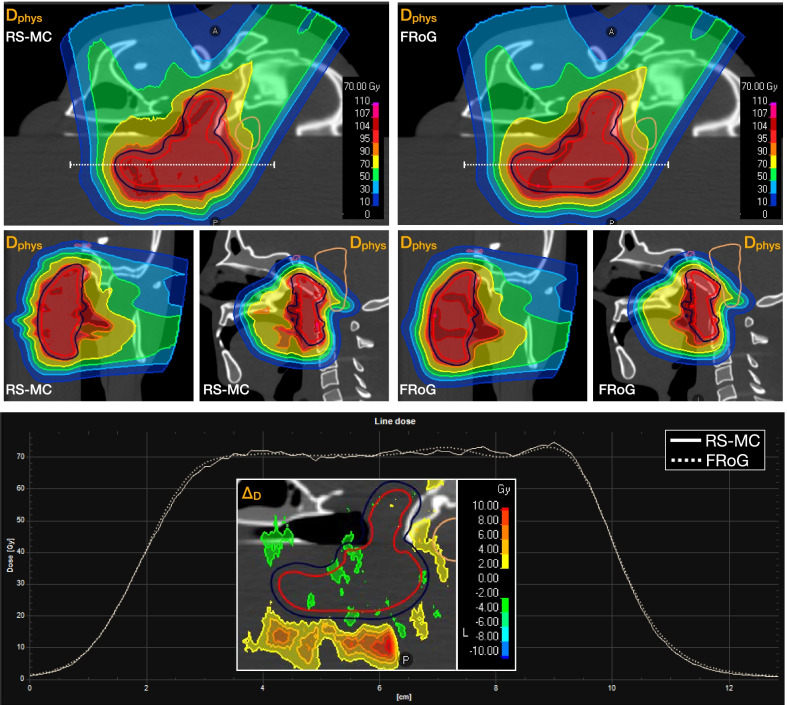
Dose calculation performance between RS-MC and FRoG was tested using an anthropomorphic head phantom with beams passing through complex heterogeneous regions within the head phantom. Dose maps and line profiles are displayed. Percent dose difference (%∆D) is provided demonstrating good agreement in the CTV while distal beam dose distortions from traversal through bone/soft-tissue/air interfaces resulted in range variations

Physical validations of FRoG in clinical-like scenarios performed using an anthropomorphic head phantom set-up yielded good agreement between FRoG and RS-MC. Figure 2 presents an overview of results for investigating performance in heterogenous regions. In summary, deviations in all DVH metrics (CTV and OAR) between FRoG and RS-MC were within <0.5% and the 3D-Γ passing rate for 3%/1.5 mm with DT10 (local) was 95.6%.

**Fig. 3:**
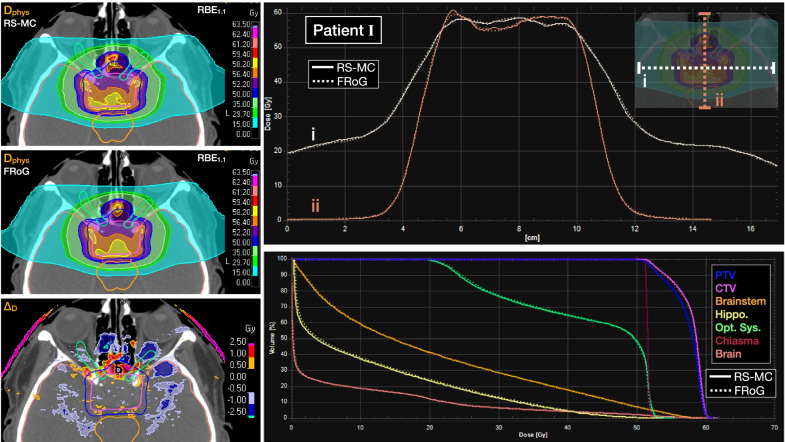
Representative calculation comparison (Patient I in Table 1) of FRoG versus RayStation (RS-MC) for a pituitary adenoma case. D_RBE_ applying fixed RBE = 1.1 for RS-MC and FRoG are displayed with ∆D_RBE_. Lateral and depth-wise dose profiles as well as dose volume histogram (DVH) plots for PTV, CTV, brainstem, hippocampus, optic system, chiasma and whole brain are provided

Across the investigated head patient cohort, mean 3D-Γ passing rates are provided in Table 2. Mean absolute percent difference in DVH metrics for CTV and OARs are additionally provided in Table 2. %∆_D_ values in the CTV were < 0.4%. For the optic nerve left and right, %∆_D_ in D_2_ was ~ 0.5%. For the brainstem and chiasma, variation in %∆_D_ or D_2_ ranged from 1 to 2%. A representative patient of the cohort (Patient I from Table 1) is presented with dose maps, line profiles and DVH comparing FRoG and RS-MC (Fig. 3). As expected, the largest global deviations occurred outside of the target volume within and in the vicinity of the nasal air cavity, which were on the order of 0.5–2% depicted in the ∆D_RBE_ map. Overall, variations between FRoG and RS-MC were well within clinical tolerances for the investigated patients.

**Table 2:** 3D-Γ analysis for investigated patient cases evaluating FRoG with RS-MC as reference, presenting mean (µ) and standard deviation (σ) in passing rate within the cohort

3D-Γ passing rates – FRoG vs. RS-MC								
Test	Type	Dose threshold (DT)	2%/2mm		3%/1.5mm		5%/1mm	
			μ	±σ	μ	±σ	μ	±σ
3D-Γ	Local	DT_10_	97.3	1.7	96.1	2.2	94.7	2.4
		DT_50_	97.8	1.9	96.8	2.5	96.0	2.4
		DT_90_	96.7	3.4	97.1	3.0	98.6	1.6
	Global	DT_10_	98.3	1.2	97.5	1.6	97.5	1.5
		DT_50_	98.0	1.9	97.2	2.3	97.1	2.0
		DT_90_	96.7	3.4	97.2	3.1	98.6	1.6

Percent derivation (%Δ) in DVH metric – FRoG vs. RS-MC								
Test	Structure	%ΔD_98_		%ΔD_50_		%ΔD_2_		
		μ	±σ	μ	±σ	μ	±σ	
DVH	CTV	0.49	0.29	0.26	0.22	0.43	0.38	
	Brainstem	–	–	0.37	0.24	1.63	2.06	
	Chiasma	–	–	0.70	0.90	1.00	1.32	
	Optic nerve (R)	–	–	0.38	0.36	0.52	0.33	
	Optic nerve (L)	–	–	2.68	3.23	0.54	0.89	

Percent mean D_RBE_ deviation in dose metrics (µ ± σ) applying fixed RBE = 1.1 between FRoG and RS-MC within the patient cohort

**Fig. 4:**
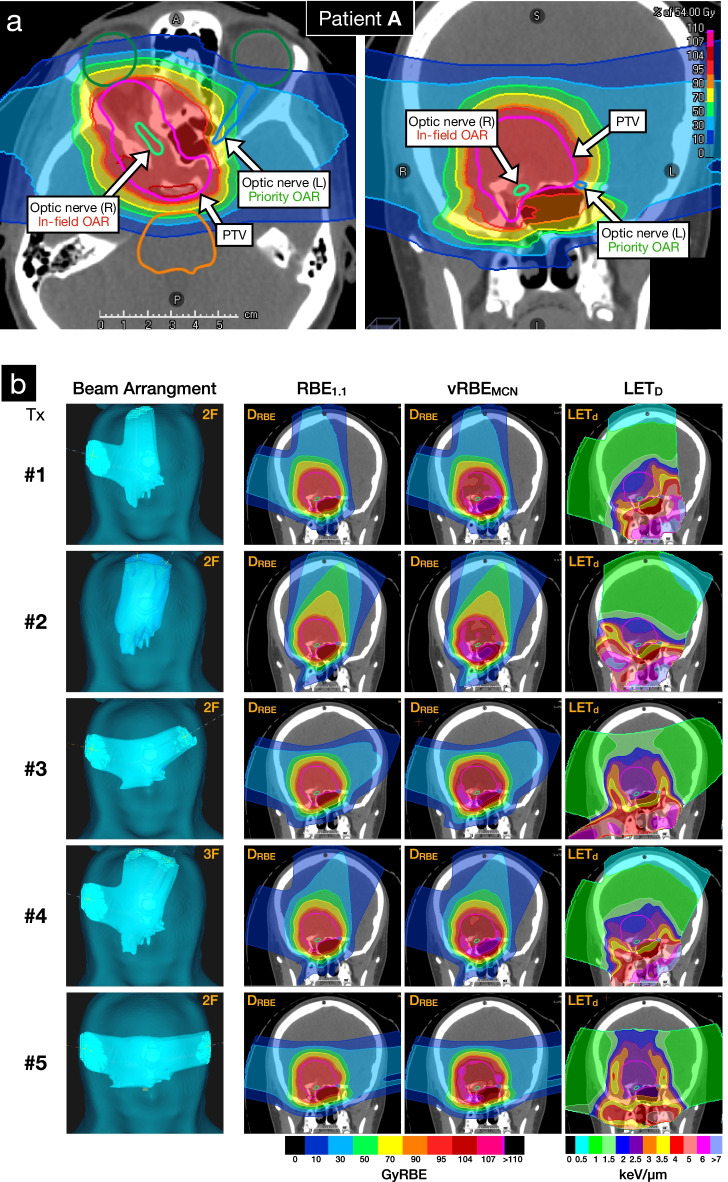

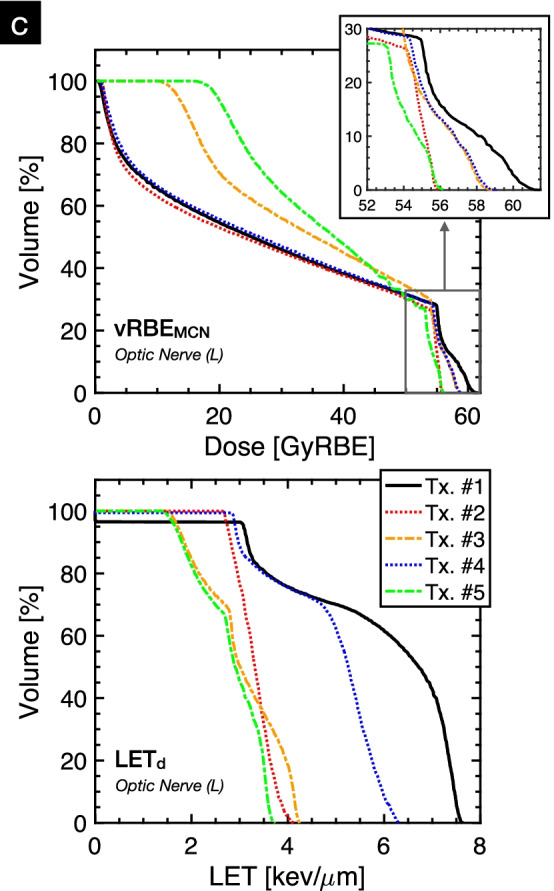
Pediatric low-grade glioma case study. treatment planning considerations for high priority sparing in the left optic nerve to preserve vision/function and reduce risk of toxicity in the ocular system (**a**). FRoG-assisted treatment design is displayed with five potential plans (Tx.#1-.#5). Beam angle arrangement for the various plans using 2 or 3 beams, clinical RBE (RBE_1.1_), variable RBE applying the McNamara et al. model (vRBE_MCN_) and LET_d_ (**b**). RBE-weighted dose volume histogram (D_RBE_VH) applying vRBE_MCN_ and LETd volume histogram (LETVH) in the contralateral optic nerve (L) for the five plans (I-V) are displayed (**c**)

Specifically for the pediatric case study (Patient A from Table 1), forward calculation in FRoG of the five treatment options (Tx. #1-#5) using a fixed RBE of 1.1 were in good agreement with RS-MC in the CTV with %∆_D50_ of 0.29(± 0.06)%. Figure 4a highlights the complexity of structure arrangement, labeling the CTV, in-field OAR (optic nerve [R]) and priority OAR (optic nerve [L]). For the contralateral optic nerve (left), FRoG deviation from RS-MC predictions for fixed RBE for D_2_ ranged from 0.04% to 2.0%, with Tx. #5 presenting the lowest deviation across the treatment options. Regarding vRBE_MCN_ predictions using FRoG, D_2_ values for Tx #1 to #5 were as follows: 60.2GyRBE, 55.6GyRBE, 58GyRBE, 58.2GyRBE and 55.6GyRBE. As for LET using FRoG, LET_2_ values for Tx #1 to #5 were as follows: 7.6 keV/µm, 4.0 keV/µm, 4.2 keV/µm, 6.2 keV/µm and 3.6 keV/µm. Treatment plan #5 was subsequently selected for patient treatment.

## Discussion

### Validation at NPTC

NPTC start-up began in Q3/2018 after machine and beam-model acceptance testing and commissioning. Verification in challenging clinical-like set-ups using various dosimetric tools, e.g., PPC05 and MatriXXOne (IBA dosimetry), took place for IMPT planning and delivery in homogenous settings (Fig. 1) and with an anthropomorphic CIRS head phantom (Fig. 2). In this scenario, the clinical TPS (RS-MC) was validated against measurements with 2D-Γ analysis. At 3%/1 mm (local) 10% dose-threshold (DT), all tests at various depth including target and distal fall-off passed with > 95% agreement, while for at 2%/1 mm (local) 10% DT, passing rates were > 90%. These results justified the consideration of RS-MC, alongside measurements, as gold-standard reference in commissioning FRoG.

Multi-institutional collaboration between particle therapy centers led to the development of an independent dose and LET_d_ engine for IBA PT facilities, beginning with the Proteus®ONE system. FRoG was successfully installed at the NPTC facility in Q1/2019 and has undergone a series of validations detailed in this work. Specific modifications to the FRoG physics beam-model were made to properly adapt the GPU-accelerated code to the Proteus®ONE system, beginning with base data composed of both physical measurements taken during NPTC commissioning and MC simulation data. With universally applicable base data generation for Proteus®ONE and implementation of the two-source VSAD approach (see *Appendix*) validated within this work, the FRoG approach can be applied to any facility hosting the Proteus®ONE system, aside from minor adjustments for facility specific definition of beam energy and foci. Compared to other centers where FRoG is in use, commissioning at NPTC involved larger RaShi thicknesses (~ 6.5 cm) and subsequent handling physics of lateral dose spread with a triple Gaussian model due to the relatively large beam modifier. Similar works detail the development of computer-driven (less user-dependent) generation of MC beam-models for scanned proton and carbon ion delivery systems [33].

**Fig. 5:**
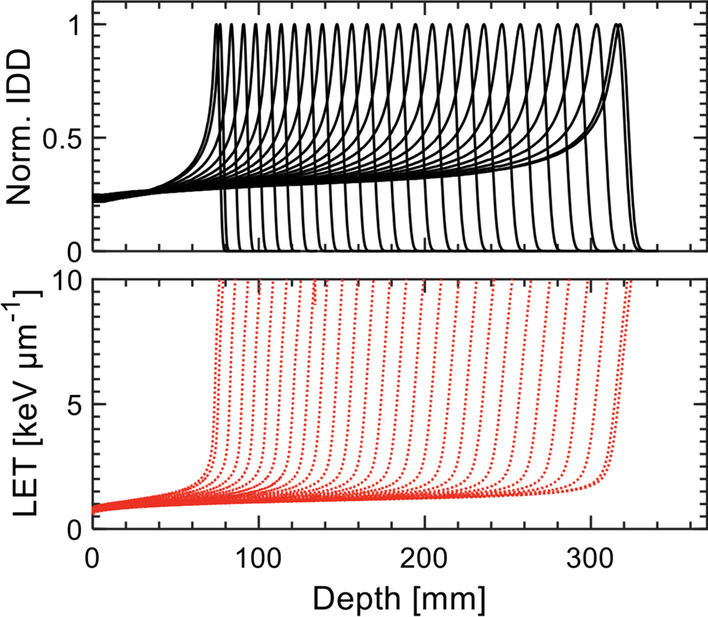
Exemplary FRoG physics database for the Proteus®ONE system, displaying 25-cm normalized integrated depth dose (IDD) and dose-weighted linear energy transfer (LET_d_) for energies ranging from 98 to 226 MeV

With respect to the dosimetric accuracy of FRoG computations for IEL and SOBP, agreement with RS-MC and measurements were well within clinical acceptability. For cases with RaShi, deviations of ~ 1.5% between FRoG and RS-MC were found for air gaps > 13 cm, due to underestimation in modeling spread in lateral dose evolution. These RaShi-to-skin distance dependent discrepancies are in line with other works using PBA in homogeneous geometries [34], however, a majority of patient treatments involve RaShi air gaps < 10 cm (Table 1), which demonstrate dose differences < 1% in SOBPs between FRoG and RS-MC.

FRoG predictions in all anthropomorphic head phantom tests with beam modifiers (RaShi), which involved oblique gantry angles with respect to the phantom surface, were in agreement with the measurement validated MC code. For example, both tests in the homogenous head region with small and large RaShi air gaps, FRoG and RS-MC were in agreement and for the most heterogeneous “base-of-skull” region, deviations were increased but with results still clinically acceptable, which was not seen previously in recent PB algorithm implementations [34–36]. Therefore, for clinically relevant cases, FRoG and RS-MC can be considered as comparable dose engines at NPTC. This was further confirmed in the 9-patient cohort where deviations were not of clinical significance.

Despite the high level of agreement between FRoG and RS-MC for the investigated cases, the use of analytical dose algorithms like FRoG may be subject to scrutiny for extreme cases which include beam modifiers (e.g., RaShi). During clinical practice, the separation distance between the RaShi face and skin surface (D_RaShi_) is minimized whenever possible and usually varies between 1.5 and 8 cm depending on the case, especially for larger thicknesses as used for Varian and IBA facilities with > 7 cm RaShi WET. For cases with D_RaShi_ < 10 cm, the effect of beam spread is quite minimal and FRoG can predict these changes quite well compared to MC calculation. Scenarios with D_RaShi_ >> 10 cm are seldom but if unavoidable, analytical systems like FRoG should be evaluated for just application. Furthermore, for extremes in high density materials (e.g., metallic implants) and low density patient anatomy (e.g., lung), FRoG shows promising results compared to MC systems [37], but it should still be understood that there are limitations in accuracy for analytical systems for such sites [38]. Small targets << 3 cm may also be problematic however this has yet to be investigated since such cases are rarely encountered at NTCP.

In all SOBPs, phantom studies and patient cases with clinically relevant RaShi positions, differences in FRoG and RS-MC target dose metrics were < 0.5%. For all DVH metrics in OARs, FRoG predictions were generally higher than RS-MC and the magnitude of the difference was dependent on indication and OAR structure, which can be attributed to the differences in modeling beam modifiers (RaShi) between an analytical algorithm and MC code. For instance, the left optic nerve was situated in dose gradients or low-dose regions, with mean and median dose between all head case plans of 19.4(± 19.7)GyRBE and < 6.7GyRBE, respectively, while the right optic nerve was in-field in most treatment cases (median D_50_ of 40.7GyRBE). This can explain in part more elevated deviations between FRoG and RS in the D_50_ for the left optic nerve. One could argue that given dose calculation uncertainties, FRoG can provide an upper bound for OAR dose constraints in general for conversative patient analysis and treatment planning.

This study was limited to investigations within head and base-of-skull patient cohort, representing the majority of patients treated at NPTC. However, additional evaluations of other localizations, e.g. thoracic and pelvic, were performed and findings are encouraging with deviation in D_mean_ in the CTV ≤ 2% for both breast and sacral tumors using RaShi. Mean D_2_ deviation in OARs situated within the dose fall-off transitioning from breast to thoracic cavity was ~ 6(± 2)%, in line with known uncertainty of modern PBA algorithms in lung [37], showing substantial improvements compared to commercial approaches [38]. Recent works using an independent MC dose engine for machine QA as well as LET_d_ and D_RBE_ computation determined treatment characteristics in OARs for breast cases at their respective facility [39]. Thorough investigations are warranted for FRoG applications beyond brain and H&N disease sites at Proteus®ONE facilities. Other facilities using FRoG for lung treatment calculations have shown clinically acceptable results in high dose regions [37].

### Pediatric case report

PT is suspected to be advantageous in place of photon treatments, particularly in pediatrics. Nonetheless, the uncertainties associated with PT in terms of beam-modeling in patients, range and set-up may put patients at risk of adverse effects. For pediatrics, it is especially important to minimize dose to normal tissues which may result in secondary cancers [40, 41] and reduce risk of toxicities (for instance, Patient A in Fig. 4a).

For Patient A, beam arrangement, RBE and LET maps of the five potential treatments (Tx.#1–5) evaluated for clinical application are provided in Fig. 4b. Tx.#5 was selected for clinical use considering the three following points: (1) among the five treatment options, for fixed RBE = 1.1, the independent calculation showed the least variation compared to RS-MC in DVH metrics for the critical structures, particularly the contralateral optic nerve (target and OAR locations highlighted in Fig. 4a), confirming robustness of the plan towards the OAR. (2) the independent engine predicted that Tx.#5 exhibited the lowest LET_2_ in the contralateral optic nerve (52% lower than Tx.#1). (3) in terms of biologically weighted dose with vRBE_MCN_, Tx.#2 and Tx.#5 yielded the lowest D_2_ in the contralateral nerve. One must note that a lower LET_d_ does not indicate a lower D_RBE_, as seen for Tx.#3 (Fig. 4.c). Considering the definition set by EPTN for optic system preservation of equivalent dose in 2 Gy fractions (EQD2) of 55 Gy at D_0.03 cc_ [42], the D_RBE_ applying McNamara et al. [32] of 56.3GyRBE corresponds to EQD2 of 54.6GyRBE, successfully meeting EPTN recommendations.

To clarify, during treatment planning, the constraints for optic system were set tighter than the clinical criteria of 55 Gy recommended by the EPTN recommendation to mitigate variable D_RBE_ enhancement at distal edge. Furthermore, applying the standard EQD2 convention for the optic nerve (with (α/β)_x_ = 2 Gy), 55.6 Gy RBE in 30 fractions for 1.87GyRBE/fx is equivalent to 53 GyRBE for 2GyRBE/fx. Therefore, the set constraints on biological dose optimization agree with fractionation schemes applied and below the EPTN limit of 55 GyRBE.

In summary, FRoG calculations for fixed and variable D_RBE_ showed that considering inter-model uncertainties both differences in the physics engines and RBE definitions, Tx.#5 predicted the greatest aptitude for robust delivery (physical and biological). The other plans which were viable and met optimization criteria requested in RayStation considering fixed RBE = 1.1 and OAR optimization goals did not predict the same level of robustness and reliability between the physics engines and biological perspectives.

From the two-year post-treatment follow-up with Patient A, no measurable toxicity effects in the left ocular nerve were observed and left eye vision was preserved. Although anecdotal, the pediatric case report presented here may guide future use of an independent dose engine for LET_d_ and/or D_RBE_ assessment (using fixed and variable RBE schemes) during clinical practice. Furthermore, this work details the first account of FRoG applications beyond retrospective study and instead within an active clinical workflow to positively impact clinical practice in reducing potential OAR toxicity without jeopardizing tumor control.

### Clinical implications

The FRoG tool could be particularly ideal for centers which transition from the photon world as a starting point for applying practical means of biologically informed decision making using fixed RBE = 1.1, variable RBE and LET_d_ analysis. As presented here, clinical support with FRoG may provide particular insight regarding facility definitions for case specific treatment delivery parameters such as beam arrangement (i.e., number of beams and angles) and consequence on LET_d_ distribution and biological dose uncertainty.

The auxiliary computations and metric analyses (LET_d_ and D_RBE_) presented here were made practically accessible via GPU-acceleration to meet the clinical pace of treatment planning (within minutes). Secondly, independent dose and LET_d_ engines may help establish protocols and gain confidence while introducing novel treatment planning procedures, e.g., from simple optimization methods like SFO to more complex techniques like MFO, which although provide increased targeting and OAR sparing, may be more prone to uncertainties. More advanced optimization protocols, e.g. LET-optimization [43], are desirable in the clinical TPS; however practically, these methods are not yet available or fully standardized for clinical use. In this study, the SFO approach was chosen to ensure homogeneous target dose distributions for each individual beam, which can reduce sensitivity to patient position and SPR uncertainties. Centers like NPTC and HIT can apply SFO for head cases during clinical practice, whenever possible, to achieve good CTV coverage while respecting OAR constraints. Other centers follow a different treatment optimization approach and find more optimal distributions for dose and LET for meeting OAR constraints using MFO. However, this technique may not inherently lead to more uniform LET distribution overall without explicitly linking to an LET optimization algorithm, which have yet to be made clinically available for standard use. Nonetheless, with systems like FRoG, centers can evaluate and scrutinize potential optimization approaches depending on desired effects (e.g., robustness, intra-field spot homogeneity, LET distribution, etc.)

In general, it is well known that uncertainties in PT have specific biophysics implications, particularly end-of-range where LET and RBE gradients may elevate potential toxicity [13]; however, without institutional feedback, e.g., treatment evaluation through clinical outcome studies in large patient cohorts, the best use of this additional information may involve qualitative interpretations. A more practical application towards quantitative use of LET and RBE distributions, as performed in this study, would involve forward calculations of treatment plan for different plan parameter settings, e.g., beam arrangement and angle selection for particular indications, tumor sizes and locations.

Currently there are more than 25 centers worldwide hosting a Proteus®ONE system. Aside from serving as a secondary dose engine for treatment planning and patient-specific QA cross-checks, systems like FRoG may serve centers which do not have a multi-purpose MC code, which requires considerable dedicated research time/budget and computational power not present at most facilities. Related works demonstrated FRoG’s capacity to function as an independent dose engine at a ProBeam® (Varian) facility for patient QA and providing dose and LET_d_ robustness analysis with patient set-up and range uncertainty [20]. IBA dosimetry does offer packages for supporting independent dose engines within the myQA iON framework [44]; however, such platforms may be limited in scope to integrate novel clinical procedures and studies as performed in the FRoG partnership.

Concurrently to its ongoing clinical application, future work at NPTC will involve FRoG during clinical trials to link possible clinical outcomes and end-points with biophysical predictors measured via dedicated MRI procedures pre-, during and post-treatment. Correlation of biophysical properties (e.g., LET_d_ and D_RBE_) with changes in anatomic, physiologic, and metabolic features in the brain is of particular interest for upcoming PT research.

Based on the results in this work, the authors encourage other facilities to request LET_d_ and variable RBE computation and optimization schemes from their TPS vendor to serve as supplementary analysis during clinical treatment design for reduction of uncertainties and potential toxicities. Future efforts in the FRoG project will transition to compatibility with beamlines from other vendors or system models.

Aside from the methods applied in the pediatric case report, there is no universal application or clear instruction for clinical integration of novel metrics like LET_d_ and variable RBE. Groups have incorporated LET_d_-based optimization techniques into their clinical TPS and would ideally be made available in all commercial TPSs [4]. Nonetheless, optimization of LET_d_ distributions alone may not be sufficient to influence the broad spectrum of biophysical uncertainties. For example, uncertainties related to tissue/cell line dependent response across the clinical LET range, demonstrated that in comparisons of various phenomenological modeling approaches, clinically relevant RBE uncertainties are present along the beam path from EC to BP [45]. Thus, a hybrid approach to treatment planning assessment using both LET_d_ and RBE may be warranted.

Recent reports discuss current and future strategies using FRoG and comparable systems to establish refined treatment planning perspectives between Nordic PT centers in the assessment of LET and/or variable D_RBE_ distributions within OARs [46]. Similarly, a beam orientation optimization technique based on LET reduction in OARs has been investigated within a research platform demonstrating the importance of such beam parameters in OAR LET reduction [47]. Nevertheless, such solutions are not available within mainstream TPS environments. Furthermore, there is no dosimetric method for clinic verification or measurement of LET. In that regard, efforts should proceed to introduce devices and protocols for validation of LET calculation systems in the clinic [48].

For the time being, auxiliary engines like FRoG are compatible with beamlines of major PT vendors and can serve as a training ground for physicians and physicists to investigate/familiarize with LET and RBE-related endpoints. In this work, a case-example for how an independent dose engine may influence clinical decision-making. FRoG continues to support clinical workflow at NPTC for best-case selection among a set of treatment options as described in the work. The FRoG network is open to centers hosting Proteus®ONE or Proteus®PLUS systems and for those interested in implementing FRoG, please write to FRoG.HIT@med.uni-heidelberg.de with details regarding intended use.

## Conclusion

An independent dose and LET_d_ engine was developed and validated for IBA Proteus®ONE systems. FRoG demonstrated good agreement with RS-MC and measurements in homogenous settings (IEL and SOBP) and in an anthropomorphic head phantom. Patient computations were assessed with RS-MC as reference, finding clinically acceptable agreement in DVH metrics in target and OARs. Lastly, a pediatric clinical case study using FRoG-assisted treatment planning was reported, advocating further efforts to establish structured yet conservative protocols for LET_d_ and D_RBE_ guided clinical practice for PT. This work demonstrates the need for further prospective and retrospective investigations on the clinical utility of additional calculation tools and metrics for best plan selection and understanding unexpected toxicities.

## Figures and Tables

**Fig. 6:**
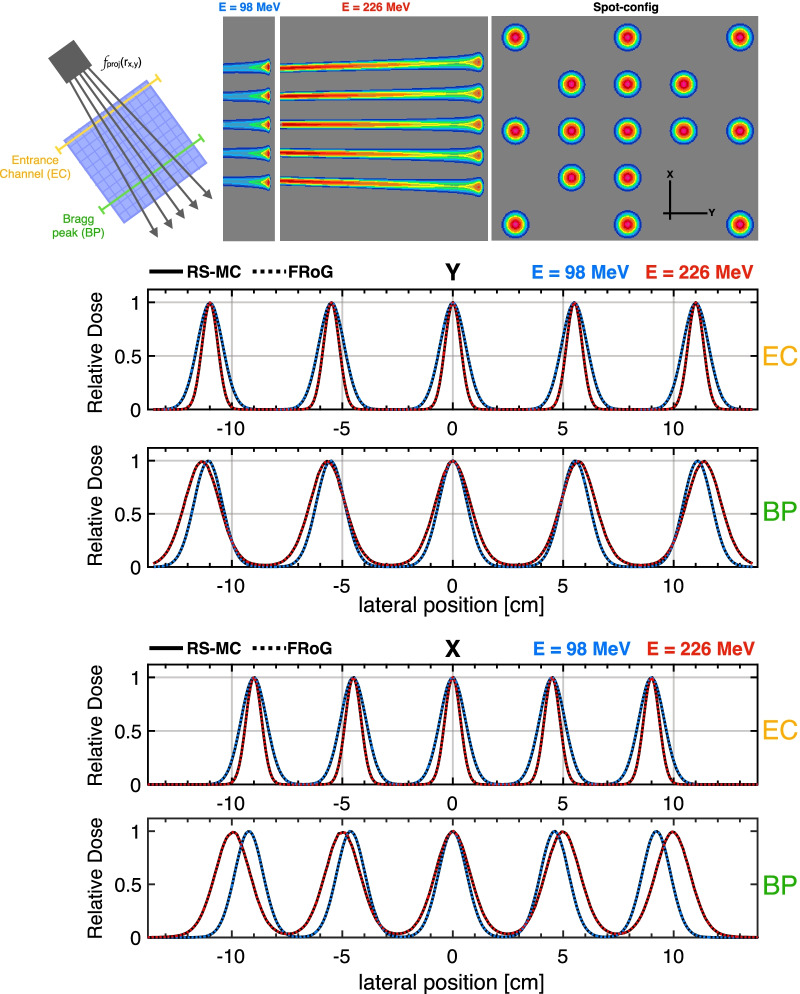
Evaluation of VSAD implementation and impact on GPU-accelerated ray tracing and dose kernel execution via beam projection (spot position and FWHM) evaluation of FRoG against reference RS-MC calculation for the 16-spot plan. The lower and upper limit of the commissioned clinical energy range (98 MeV and 226 MeV) are tested to demonstrate depth and energy dependence of beam propagation. X and Y line profiles are presented for entrance channel (EC) and Bragg peak (BP)

## Data Availability

Research data are stored in an institutional repository and will be shared upon request to the corresponding author.

## Appendix

### FRoG beam-model for the Proteus®ONE system

FRoG was installed at NPTC on an Intel Core i7 I7-7700 K (4.2 GHz, 16 GB RAM) with a NVIDIA GTX 1080Ti graphics card. In addition to previous modifications made for the Varian ProBeam facility, the FRoG dose engine was updated according to specific requirements of the Proteus®ONE system. Such cyclotron-based delivery systems operate using continuous selection (as opposed to discrete energies with a synchrotron) and beam characteristics differ between offered treatment room models as well as vendors using similar equipment. First, standard commissioning data from the facility start-up, e.g., integral depth dose (IDD) profiles, dose calibrations with iso-energy layers (IELs) and single spot-sizes in air measured from -34 cm to + 100 cm from isocenter via the StingRay, parallel-plate ionization chamber (PPC05) and Lynx (IBA Dosimetry), respectively, were compiled to generate the FRoG beam model for the Proteus®ONE system. The relative IDDs acquired via the StingRay were converted to absolute dose by scaling each curve by the dose calibration measurements acquired with the PPC05 at 3 cm depth in water for a 10.4 × 10.4 cm2 field, with a 2 mm spot distance, for each IEL. Measurements were conducted for 28 proton beam energies ranging from 98.4 MeV to 226 MeV, corresponding to Bragg peak positions (R_80_) of ~7.5 to ~32 cm. Due to the large RaShi thickness used at the NPTC (~ 6.5 cm Lexan, with water-equivalent thickness of ~ 7.4 cm), a higher order Gaussian beam model was parameterized using a triple Gaussian to describe the lateral dose evolution in water and the low-dose envelope (secondary beam profile from scattering in the nozzle and beam modifiers). FLUKA MC simulations were conducted to generate the corresponding IDDs and lateral beam evolution data used for parameterization. TG parameterizations are obtained by fitting radially scored FLUKA dose distributions as a function of depth in water for all commissioned energies. To use facility specific IDD measurements as FRoG base input, universal Proteus®ONE beam corrections were devised to adjust StingRay measurements accounting for lost dose contribution outside of the detector. IDD simulation in FLUKA MC scored dose radially with radii of 6 cm (StingRay integration size) and 25 cm. Subsequent IDDs ratio between the 6 cm and 25 cm simulation sets was used as depth-wise scaling factors for the measured profiles to use as FRoG’s input. Integral depth LET_d_ for Z = 1 particles was additionally scored radially using the same 25 cm radius condition. For eventual evaluation of LET_d_ distributions and proper implementation of the McNamara variable RBE model, LET was specifically defined by only scoring Z = 1 particles (primary and secondary protons) in the FRoG database generation of LET_d_. In sum, for the commissioned energies at NPTC, a database containing IDD, LET_d_ (Fig. 5) and TG parameters (sigma (*σ*) and weight [*w*]) were generated for beams without modifiers. For beams with RaShi, specific TG databases were generated for RaShi to skin distances between 1.5 and 43 cm with step-size of 1.5 cm. During the calculation process in FRoG, a database is interpolated online for the planned energies (and if present, at a specified RaShi-to-skin distance) using the reference databases. Dose in FRoG is scored within a circle around every pencil with a radius of 3 × σ(x), where σ(x) is the maximum Gaussian standard deviation of the TG beam shape approximation at depth x in water.

### Dual virtual source axis distance (dVSAD) implementation and verification

As opposed to passive scattering which relies on beam modifiers and collimators for beam shaping, active beam delivery with raster-scanning particle therapy technology uses two perpendicular magnets to steer the beam to deliver complex geometric shaped dose distribution to the tumor volume with high-precision [30]. These steering magnets are situated upstream from the exit window and their beam deflection effects are modeled in the TPS by two virtual “source” points to effectively account for both the inherent beam divergence and position of the scanning magnets.

Initial development of FRoG took place for beam delivery systems which have similar positions and distances from isocenter for the x and y-coordinate scanning magnets used for 2D pencil beam scanning. For instance, at HIT the virtual positions of the scanning magnets before the beam application and monitoring system, are ~ 6.8 m and ~ 42 m upstream from isocenter for fixed-beam and gantry treatment rooms, respectively. Specifically for the fixed-beam room, VSAD_x,INST-HIT_ = 7.2 m, VSAD_y,INST-HIT_ = 6.5 m and therefore, an average VSAD (${\underline{VSAD}}_{x,y}$) is approximated. Similarly for the Varian ProBeam® center, the x- and y-coordinate scanning magnets positions in HIT were approximated by a single point using ${\underline{VSAD}}_{x,y}$ [20].

In contrast, ∆VSAD_x,y_ =|VSAD_x_–VSAD_y_| for Proteus®ONE beam was not < <${\underline{VSAD}}_{x,y}$ (VSAD_x,IBA_ = 2.9 m, VSAD_y,IBA_ = 9.7 cm) and could not be approximated as a single point source. Therefore, the positions of beam deflection in the two scanning magnets are taken into account by an y-rotation, followed by a distal x-rotation. For pencil beams along the central axis (CAX), VSAD handling procedures do not impact raytracing and in turn dose computation. However, for off-axis pencil beams (∆_x,y_ ≠ 0), approximating the scanning magnets with a single point source will improperly set the entrance angle for each raytrace and the magnitude of the deviation in beam propagation will increase with ∆_x,y_. For instance, the ${\underline{VSAD}}_{x,y}$ approximation at ∆_x,y_ = 10 cm at isocenter would result in an x- and y-angular deviation of ~ 1° and ~ 0.3° during raytracing and beam propagation. Although the effect of < 1° may be relatively minor for small ∆_x,y_ and beams with lower particle energy/range, the ${\underline{VSAD}}_{x,y}$ approximation could yield large deviations in the Bragg peak position of >5 mm for the highest energy. In turn, the FRoG dose engine components for GPU-accelerated ray tracing and in turn, dose kernel execution were modified to account for x,y-dependency of VSAD for the Proteus®ONE system (VSAD_x_ ≠ VSAD_y_).

To verify implementation of the IBA Proteus®ONE beam-model prior to dosimetric investigations, and in particular the dVSAD handling, benchmark tests with low, mid and high energies were performed using a complex grid of 16 individual spots covering the scanning field of view (20 × 24cm^2^) for the Proteus®ONE system (Fig. 6). Entrance and BP spot shape and position were validated against the reference MC system used for clinical treatment planning (RS-MC). For instance, Fig. 6 depicts proper implementation of dVSAD for the 96 MeV and 226 MeV (minimum and maximum proton beam energies, respectively).

### Validations using an anthropomorphic head phantom

Two studies to compare calculation performance of FRoG and RS-MC in homogenous and heterogenous scenarios using an anthropomorphic head phantom (CIRS PT Dosimetry Head, Model 731-HN) were performed. During facility commissioning, RS-MC prediction was extensively validated in absolute dose measurements using the MatriXXOne 2D ion chamber array (IBA dosimetry). The first study investigated FRoG calculation performance in the homogeneous brain region of the phantom (single field optimization [SFO] with beam angles 0°/45°) using a clinically representative air gap of ~ 6 cm between RaShi and the skin, as well as an extreme air gap distance of ~ 32 cm. A second test evaluated accuracy of dose prediction in the highly heterogeneous region (H&N/skull-base with several interfaces between air cavities and surrogates for bone and soft-tissue) for a RaShi air gap of ~ 5 cm (multiple field optimization [MFO] with beam angles 35°/345°).

For tests in the homogenous head region and d_RaShi_ ≈ 6 cm, differences in dose volume histogram (DVH) metrics D_98_, D_50_, D_2_ and D_1_ between FRoG and RS-MC were all <0.3% in the CTV. For testing more extreme RaShi to skin distance (d_RaShi_ ≈ 32 cm) within the same homogenous head region, differences in D_98_, D_50_, D_2_ and D_1_ rose to 1.4%, 1.06%, 0.1% and 0.6%, respectively, demonstrating the capacity of FRoG’s analytical approach to accurately model physics of beam modifiers, relatively comparable to MC. With RS-MC as reference, the mean 3D-Γ passing rate for all tests with FRoG in the homogenous head region was 99.2(± 1.09)%.

In the heterogeneous H&N region, all CTV DVH metrics for FRoG and RS-MC were within <0.4%. Deviations in D_2_ and D_1_ for the brainstem and chiasma structures were both <0.5%. The largest calculated deviations were D_2_ and D_1_ in the left optic nerve, with %∆ of 1.3% and 1.1%, respectively. Line profiles and dose difference maps are additionally provided in Fig. 2 of the main text. With RS-MC as reference, the 3D-Γ passing rate for 3%/1.5 mm with DT10 (local) was 95.6% and the mean passing rate for all 3D-Γ tests in the heterogenous head region was 93.8(± 2.96)%. 2D-Γ passing rate (local) for RS-MC and MatriXXOne measurements at various depths for 3%/1 mm with DT10 were all >95%. Results are summarized in Fig. 2 of the main text.

## Abbreviations

CTV: Clinical target volume
FRoG: Fast Robust dose Engine on GPU
GPU: Graphics Processing Unit
HIT: Heidelberg Ion-beam Therapy Center
IEL: Iso-energy layer
IMPT: Intensity Modulated Proton Therapy
LET: Linear Energy Transfer
NPTC: Normandy Proton Therapy Center
OAR: Organ-at-risk
PT: Proton Therapy
RaShi: Range Shifter
RBE: Relative Biological Effectiveness
SOBP: Spread-out Bragg peak
QA: Quality Assurance
VSAD: Virtual source axis distance

## References

[1] Newhauser WD, Zhang R (2015). The physics of proton therapy. Phys Med Biol..

[2] Mohan R, Grosshans D (2017). Proton therapy - Present and future. Adv Drug Deliv Rev..

[3] PTCOG - Facilities in Operation. available at: https://www.ptcog.ch. Accessed Dec 2021.

[4] Unkelbach J, Paganetti H (2018). Robust proton treatment planning: physical and biological optimization. Semin Radiat Oncol..

[5] Liu C, Patel SH, Shan J (2020). Robust optimization for intensity modulated proton therapy to redistribute high linear energy transfer from nearby critical organs to tumors in head and neck cancer. Int J Radiat Oncol Biol Phys.

[6] An Y, Shan J, Patel SH (2017). Robust intensity-modulated proton therapy to reduce high linear energy transfer in organs at risk. Med Phys.

[7] Chaudhary P, Marshall TI, Perozziello FM (2014). Relative biological effectiveness variation along monoenergetic and modulated Bragg peaks of a 62-MeV therapeutic proton beam: a preclinical assessment. Int J Radiat Oncol Biol Phys.

[8] Paganetti H, Niemierko A, Ancukiewicz M (2002). Relative biological effectiveness (RBE) values for proton beam therapy. Int J Radiat Oncol Biol Phys.

[9] Paganetti H (2014). Relative biological effectiveness (RBE) values for proton beam therapy. Variations as a function of biological endpoint, dose, and linear energy transfer. Phys Med Biol.

[10] McMahon SJ, Paganetti H, Prise KM (2018). LET-weighted doses effectively reduce biological variability in proton radiotherapy planning. Phys Med Biol.

[11] Peeler CR, Mirkovic D, Titt U (2016). Clinical evidence of variable proton biological effectiveness in pediatric patients treated for ependymoma. Radiother Oncol.

[12] Bahn E, Bauer J, Harrabi S (2020). Late contrast enhancing brain lesions in proton-treated patients with low-grade glioma: clinical evidence for increased periventricular sensitivity and variable RBE. Int J Radiat Oncol Biol Phys.

[13] Paganetti H, Blakely E, Carabe-Fernandez A, et al. Report of the AAPM TG-256 on the relative biological effectiveness of proton beams in radiation therapy. Med Phys. 2019;46(3). 10.1002/mp.13390 10.1002/mp.13390 PMC9559855 30661238

[14] Paganetti H, Beltran CJ, Both S (2020). Roadmap: proton therapy physics and biology [published online ahead of print, 2020 Nov 23]. Phys Med Biol..

[15] Huang S, Kang M, Souris K (2018). Validation and clinical implementation of an accurate Monte Carlo code for pencil beam scanning proton therapy. J Appl Clin Med Phys.

[16] Deng W, Younkin JE, Souris K (2020). Technical Note: Integrating an open source Monte Carlo code “MCsquare” for clinical use in intensity-modulated proton therapy. Med Phys.

[17] Senzacqua M, Schiavi A, Patera V, et al. A fast - Monte Carlo toolkit on GPU for treatment plan dose recalculation in proton therapy. J Phys Conf Ser; 2017. 10.1088/1361-6560/aa8134 28873069

[18] Mein S, Choi K, Kopp B (2018). Fast robust dose calculation on GPU for high-precision 1H, 4He, 12C and 16O ion therapy: the FRoG platform. Sci Rep.

[19] Choi K, Mein S, Kopp B (2018). FRoG—a new calculation engine for clinical investigations with proton and carbon ion beams at CNAO. Cancers (Basel).

[20] Kopp B, Fuglsang Jensen M, Mein S (2020). FRoG: An independent dose and LETd prediction tool for proton therapy at ProBeam® facilities. Med Phys..

[21] Kopp B, Mein S, Dokic I (2020). Development and validation of single field multi-ion particle therapy treatments. Int J Radiat Oncol Biol Phys..

[22] van de Water S, Safai S, Schippers JM, Weber DC, Lomax AJ (2019). Towards FLASH proton therapy: the impact of treatment planning and machine characteristics on achievable dose rates. Acta Oncol..

[23] Inaniwa T, Kanematsu N, Noda K (2017). Treatment planning of intensity modulated composite particle therapy with dose and linear energy transfer optimization. Phys Med Biol.

[24] Guterres Marmitt G, Pin A, Ng Wei Siang K (2020). Platform for automatic patient quality assurance via Monte Carlo simulations in proton therapy. Phys Med.

[25] Meijers A, Guterres armitt G, Ng Wei Siang K (2020). Feasibility of patient specific quality assurance for proton therapy based on independent dose calculation and predicted outcomes. Radiother Oncol.

[26] Aitkenhead AH, Sitch P, Richardson JC (2020). Automated Monte-Carlo re-calculation of proton therapy plans using Geant4/Gate: implementation and comparison to plan-specific quality assurance measurements. Br J Radiol.

[27] Beltran C, Tseung HWC, Augustine KE (2016). Clinical implementation of a proton dose verification system utilizing a GPU accelerated Monte Carlo engine. Int J Part Ther.

[28] Mein S, Kopp B, Tessonnier T (2019). Dosimetric validation of Monte Carlo and analytical dose engines with raster-scanning 1H, 4He, 12C, and 16O ion-beams using an anthropomorphic phantom. Phys Med.

[29] Mein S, Dokic I, Klein C (2019). Biophysical modeling and experimental validation of relative biological effectiveness (RBE) for 4He ion beam therapy. Radiat Oncol.

[30] Haberer T, Debus J, Eickhoff H (2004). The heidelberg ion therapy center. Radiother Oncol.

[31] Low DA, Harms WB, Mutic S (1998). A technique for the quantitative evaluation of dose distributions. Med Phys.

[32] McNamara AL, Schuemann J, Paganetti H (2015). A phenomenological relative biological effectiveness (RBE) model for proton therapy based on all published in vitro cell survival data. Phys Med Biol.

[33] Fuchs H, Elia A, Resch AF (2020). Computer-assisted beam modeling for particle therapy. Med Phys.

[34] Widesott L, Lorentini S, Fracchiolla F (2018). Improvements in pencil beam scanning proton therapy dose calculation accuracy in brain tumor cases with a commercial Monte Carlo algorithm. Phys Med Biol.

[35] Branco D, Taylor P, Zhang X (2017). An anthropomorphic head and neck quality assurance phantom for credentialing of intensity-modulated proton therapy. Int J Part Ther.

[36] Wang P, Tang S, Taylor PA (2019). Clinical examination of proton pencil beam scanning on a moving anthropomorphic lung phantom. Med Dosim.

[37] Magro G (2021). GPU-accelerated dose engine meets Monte Carlo accuracy in lung. Phys Med.

[38] Taylor PA, Kry SF, Followill DS (2017). Pencil beam algorithms are unsuitable for proton dose calculations in lung. Int J Radiat Oncol Biol Phys.

[39] Liu C, Zheng D, Bradley JA (2020). Incorporation of the LETd-weighted biological dose in the evaluation of breast intensity-modulated proton therapy plans. Acta Oncol (Madr).

[40] Merchant TE (2009). Proton beam therapy in pediatric oncology. Cancer J.

[41] Weber DC, Habrand JL, Hoppe BS (2018). Proton therapy for pediatric malignancies: fact, figures and costs. A joint consensus statement from the pediatric subcommittee of PTCOG, PROS and EPTN. Radiother Oncol.

[42] Lambrecht M, Eekers DBP, Alapetite C (2018). Radiation dose constraints for organs at risk in neuro-oncology; the European Particle Therapy Network consensus. Radiother Oncol.

[43] Unkelbach J, Botas P, Giantsoudi D (2016). Reoptimization of intensity modulated proton therapy plans based on linear energy transfer. Int J Radiat Oncol Biol Phys.

[44] IBA Dosimetry. myQA iON - Patient QA | IBA Dosimetry.

[45] Rørvik E, Fjæra LF, Dahle TJ (2018). Exploration and application of phenomenological RBE models for proton therapy. Phys Med Biol.

[46] Toma-Dasu I, Dasu A, Vestergaard A (2020). RBE for proton radiation therapy–a Nordic view in the international perspective. Acta Oncol (Madr).

[47] Gu W, Ruan D, Zou W (2021). Linear energy transfer weighted beam orientation optimization for intensity-modulated proton therapy. Med Phys.

[48] Gehrke T, Burigo L, Arico G, et al. Energy deposition measurements of single 1H, 4He and 12C ions of therapeutic energies in a silicon pixel detector. J Instrum 2017.

